# Serum Omega-6/Omega-3 Ratio and Risk Markers for Cardiovascular Disease in an Industrial Population of Delhi

**DOI:** 10.4236/fns.2013.49A1015

**Published:** 2013-09

**Authors:** Ruby Gupta, Ramakrishnan Lakshmy, Ransi Ann Abraham, Kolli Srinath Reddy, Panniyammakal Jeemon, Dorairaj Prabhakaran

**Affiliations:** 1 South Asia Network for Chronic Disease, New Delhi, India; 2 Center for Chronic Disease Control, New Delhi, India; 3 Department of Cardiac Biochemistry, AIIMS, New Delhi, India; 4 Public Health Foundation of India, New Delhi, India

**Keywords:** Omega Fatty Acids, Dyslipidemia, Omega 6/3 Ratio, CHD, hs CRP

## Abstract

High omega-6/omega-3 ratio intake promotes development of many chronic diseases. Secondary prevention studies though have demonstrated a decline in progression of many such diseases after reducing the intake, specific biochemical indices of cardiovascular disease risk markers have not been evaluated. We have evaluated the circulating levels of omega-6/omega-3 ratio and its effect on cardiovascular risk markers in India. Present study was conducted in industrial setting where employees were randomly selected. Data on their demographic characteristics were collected using pre-tested questionnaire. Fasting blood samples were collected from all the participants. Serum was separated and stored at −80°C till the time of analysis. Lipids were estimated using standard kits. Fatty acids in serum were estimated by Gas chromatography. The identified Omega-3 fatty acid included were 18:3 (Alpha-linolenic acid), 20:5 (Eicosapentenoic acid) & 22:6 (Docosahexenoic acid). Among omega-6 included were 18:2 (linoleic acid), 18:3 (gamma-linolenic acid) & 20:4 (Arachidonic acid). Complete data was available for 176 participants (89% males and 11% females) with mean age of 47.23 ± 6.00 years. The bmi of the participants was 24.88 ± 3.43 Kg/m^2^ and waist circumference was 91.50 ± 9.56 cm. The median of omega-6/omega-3 ratio in the study population was 36.69 (range: 6.21 - 183.69). The levels of total cholesterol, triglycerides, ldl-cholesterol and cholesterol/hdl ratio and apo B correlated significantly with omega-6/3 ratio. There was no correlation observed with hsCRP and LDL-particle size. A direct relationship of omega-6/omega-3 ratio with dyslipidemia was observed in our study.

## 1 Introduction

A balance between omega-6/omega-3 of 1/1 existed in the food of all the wild animals and human beings for millions of years. Since last 10,000 years with the growth of agriculture there is a rapid change in food habits of human being and a drastic change observed in the last 150 years with rapid industrialization and urbanization [1]. It has resulted in increased uptake of high energy food and lesser expenditure of energy (low physical activity), high intake of omega-6 fatty acids (from cereals and grains) and less intake of omega-3 fatty acid resulting in high omega-6/omega-3 ratio. Intake as high as 15 has been reported from UK & north Europe and 16 - 74 from US as compared to 1 - 2 from Greece (before year 1960) and 4 from Japan. However, the WHO recommendation of omega-6/omega-3 ratio is 5 - 8 which should form 1% - 2% of total energy intake per day [2].

A very high omega-6/omega-3 ratio promotes pathogenesis of many chronic diseases like cardiovascular disease, cancer, inflammatory and autoimoune disease, rheumatoid arthritis, asthma, whereas increased intake of omega-3 fatty acids (reduced omega-6/omega-3 ratio) suppresses the effect [3]. Secondary prevention studies have demonstrated a decline in disease progression or reversal of symptoms with decline in ratio; however the optimal level varies with the disease under consideration [4].

Relatively high intake of omega-6/omega-3 ratio of 38 - 50 and 5 - 6.1 has been reported from urban and rural India respectively [5,6]. There has always been emphasis to maintain a lower ratio of omega-6/omega-3 in the dietary intake on the basis of studies evaluating the effects of dietary n-3 PUFA on biochemical indices of CHD risk [7] and an improvement in Insulin resistance [8,9]. With the advancement of time, there is an increase in urbanization and a rapid change in food habits, there is also an accumulation of risk markers resulting in multifactorial diseases like CVD to manifest earlier in life. We did not find any study which has evaluated the circulating levels of omega-6/omega-3 ratio and affected cardiovascular risk markers in India.

## 2 Material & Methods

The present study was conducted in industrial setting where employees were randomly selected. Individuals with known history of diabetes, heart disease, stroke, chronic renal failure, thyroid abnormalities or cancer were not included for the study. Also individuals who reported having taken during the previous 3 months dietary supplements or medications known to alter lipid or glucose metabolism (for example, plant sterol-enriched foods or supplements, fish oil supplements, lipid-lowering drugs, steroids, *β*-blockers, insulin or oral hypoglycaemic drugs) were excluded from the study. Informed consent was taken from all the participants. Ethical clearance for the present study was obtained from the institutes. Data on their demographic characteristics were collected using pre-tested questionnaire. Fasting blood samples were collected from all the participants in plain tube using vacutainers from BD. Serum was separated and stored at −80°C till the time of analysis.

Cholesterol, triglycerides, HDL-c, LDL-c, Apo-A_1_, Apo-B was estimated using kits from Sentinel Diagnostic (Milan, Italy) on CX9 autoanalyzer from Beckman Coulter. hsCRP was estimated by latex enhanced immune-turbidimetry. LDL-particle size was estimated using polyacrylamide slab gel electrophoresis [10]. Fatty acids were estimated in serum by Gas chromatography as a marker of dietary fatty acid intake in the last few days using method of Leepage & Roy, 1986 [11]. Total 31 fatty acids were identified using standards from Supelco. Fatty acids are expressed as percentage of total fatty acids identified. The identified omega-3 fatty acid included were 18:3 (Alpha-linolenic acid), 20:5 (Eicosapentenoic acid, epa) & 22:6 (Docosahexenoic acid, dha). Among omega-6 included were 18:2 (linoleic acid), 18:3 (gamma-linolenic acid) & 20:4 (Arachidonic acid).

Statistical analysis was carried using STATA 9.0 (College station, Texas, USA). The strength of relationship between lipid profile and fatty acids ratio were calculated using pearson/spearman rank correlation whichever was appropriate for the distribution of the data. p value less than 0.05 were considered statistically significant.

## 3 Results

Complete data was available for 176 participants (89% males and 11% females) with mean age of 47.23 ± 6.00 years. The mean body mass index (BMI) of the participants was 24.88 ± 3.43 Kg/m^2^ and mean waist circumference was 91.50 ± 9.56 cm (Table 1). The median of omega-6/omega-3 ratio in the study population was 36.69 (range: 6.21 - 183.69) (Table 2). High levels of omega-6/3 ratio associated with increased total cholesterol, triglycerides, LDL-cholesterol, cholesterol/hdl-c ratio and apo B levels (Table 3). There was no correlation observed with hsCRP, LDL- particle size, abdominal obesity and body mass index. On regression analysis, omega-6/omega-3 ratio was significantly associated with cholesterol/hdl-c ratio and remained significant even after adjustment with non modifiable risk markers.

Omega-3 fatty acids when analyzed separately were associated with low levels of total cholesterol and cholesterol/hdl ratio. Individually, high levels of alpha-linoleic acid (ALA) correlated with lower BMI and higher levels of epa + dha correlated significantly with lower levels of cholesterol, triglycerides, cholesterol/hdl-c ratio and apo B levels. The relationship of ALA with BMI and epa + dha with chol/HDL ratio was significant on regression analysis and remained significant even after adjustment with age and sex.

Among omega-6 fatty acids, Linoleic acid (18:2) was inversely associated with triglyceride levels (r = −0.38, p = 0.02). The hypotriglyceridemic effect of linoleic acid was found significant on regression and remained significant after adjustment with other non-modifiable risk factors.

## 4 Discussion

Linoleic acid (18:2) of the omega-6 and linolenic acid (18:3) of omega-3 family are known as essential fatty acids (EFA) because human beings cannot synthesize these fatty acids and must obtain them from diet. These fatty acids in the human body undergo elongation and desaturation procedure to produce very-long chain fatty acids. There is competition between omega-6 and omega-3 fatty acids for the desaturation enzyme. However, both D-4 and D-6 desaturases prefer omega-3 to omega-6 fatty acids [12,13]. A high LA intake interferes with the desaturation and elongation of ALA [14,15]. Mammalian cells cannot convert omega-6 to omega-3 fatty acids because they lack the converting enzyme, omega-3 desaturase.

The direct relationship of omega-6/omega-3 ratio with dyslipidemia observed in our study was not found else-where in literature except for one wherein subjects were given a combination omega-3 PUFA from fish oil and plant sterols resulted in reduction in cholesterol/hdl-c ratio [16]. High prevalence (40.2 % in men & 34.4% in women) [17] and early appearance (between the age 31 - 40 years) of dyslipidemia (cholesterol/hdl-c ratio ≥ 4.5) puts Indians at risk of developing coronary artery disease early in life [18]. A balance in omega-6/omega-3 intake has always been advocated for optimal health. Apart from increase in omega-3 intake by the vegetarians in the form of supplement, there is a need of reduction in absolute omega-6 fatty acid intake.

We did not find any association with omega-6/omega-3 ratio with hs CRP. Dietary supplementation of low fat high complex carbohydrate diet supplemented with omega-3 fatty acid for 12-weeks did not result in any significant change in CRP in LIPGENE study [19].

A review of placebo-controlled, crossover or parallel design studies by Harris WS, 1997 [20] revealed that both omega-6 and ALA (omega-3) polyunsaturated fatty acids are equivalent in their effect on lipid & lipoproteins. Kinetics studies have shown that omega-3 fatty acids exert their hypocholesterolemic effect through lowering of apo B synthesis thereby reducing conversion of VLDL to LDL [21]. However, omega-6 fatty acids on the other hand exert hypolipidemic effects through increasing LDL catabolism. We observed a cholesterol lowering effect of omega-3 fatty acid which also has an impact on the chol/hdl-c ratio.

The preventive effect of epa & dha has been identified by studies since seventies in Greenland Eskimos [22], through Seven country study [23]. The significant inverse association of epa + dha with triglyceride levels in our study possibly contributes through increased clearance of chylomicron triglyceride by increasing LPL activity [24].

The major limitation of the study was that information on dietary intake was not available which would have been extremely useful in the strengthening the correlations especially percent fat and carbohydrate intake which are direct determinants of circulating triglyceride levels.

## 5 Conclusion

There is a highly skewed omega-6/omega-3 ratio intake in the study population and its strong association with dyslipidemia suggests disturbances in lipid metabolism, which need to be further evaluated.

## Figures and Tables

**Table 1:** Baseline characteristics of the study population.


Numbers	176
Mean age (years)	47.23 ± 6.00
Sex (%)	89% Males
Mean waist circumference (cm)	91.5 ± 9.56
Mean BMI (Kg/m^2^)	24.88 ± 3.43
Smokers (%)	38 (21.6%)
Alcohol intake (%)	62 (35%)
Mean Systolic Blood Pressure (mmHg)	127.39 ± 15.65
Mean Diastolic Blood Pressure (mmHg)	81.10 ± 10.46


**Table 2:** Biochemical Profile & fatty acid levels of the study participants.

Parameters	Mean SD/Median (range)
Cholesterol (mg/dl)	194.66 ± 30.70^a^
Triglycerides (mg/dl)	139.52 ± 62.35^a^
HDL-c (mg/dl)	37.47 ± 9.31^a^
LDL-c (mg/dl)	130.76 ± 28.69^a^
Cholesterol/HDL ratio	5.37 ± 1.00^a^
Apolipoprotein A_1_ (mg/dl)	125.77 ± 23.72^a^
Apolipoprotein B (mg/dl)	106.93 ± 20.01^a^
Hs CRP (mg/l)	1.7 (0.01 - 100)^b^
LDL-particle size (nm)	26.96 ± 0.49^a^
Alpha-linolenic acid (*ω*-3)	0.085 (0.01 - 2.43)^b^
Eicosapentenoic acid (*ω*-3)	0.49 (0.05 - 2.44)^b^
Docosahexenoic acid (*ω*-3)	0.54 (0.02 - 2.76)^b^
Linoleic acid (*ω*-6)	31.80 ± 5.05^a^
Gamma-linolenic acid (*ω*-6)	0.036 (0.01 - 1.89)^b^
Arachidonic acid (*ω*-6)	5.42 ± 1.72^a^
Omega-6/Omega-3 ratio	36.69 (6.21 - 183.69)^b^

a Values are expressed as Mean ± SD;

b Values are expressed as Median (range).

**Table 3:** Correlation of omega-6/omega-3 ratio with bio-chemical risk markers.

Risk markers	Correlation coefficient	p value
Cholesterol	0.203	**0.02**^c^
Triglycerides	0.187	**0.04**^c^
HDL-c	−0.071	0.44
LDL-c	0.189	**0.03**^c^
Cholesterol/HDL-c ratio	0.27	**0.002**^c^
Apo A1	−0.037	0.42
Apo B	0.204	**0.02**^c^
LDL-size	−0.04	0.66
hsCRP	−0.077	0.40

c Values are significant, p < 0.05.
